# Advanced Micro/Nanostructures for Lithium Metal Anodes

**DOI:** 10.1002/advs.201600445

**Published:** 2017-02-16

**Authors:** Rui Zhang, Nian‐Wu Li, Xin‐Bing Cheng, Ya‐Xia Yin, Qiang Zhang, Yu‐Guo Guo

**Affiliations:** ^1^ Beijing Key Laboratory of Green Chemical Reaction Engineering and TechnologyDepartment of Chemical EngineeringTsinghua UniversityBeijing100084P. R. China; ^2^ CAS Key Laboratory of Molecular Nanostructure and NanotechnologyBeijing National Laboratory for Molecular SciencesInstitute of ChemistryChinese Academy of Sciences (CAS)Beijing100190P. R. China; ^3^ School of Chemistry and Chemical EngineeringUniversity of Chinese Academy of SciencesBeijing100049P. R. China

**Keywords:** anode, batteries, Li metal, nanostructure, solid electrolyte interphase

## Abstract

Owning to their very high theoretical capacity, lithium metal anodes are expected to fuel the extensive practical applications in portable electronics and electric vehicles. However, unstable solid electrolyte interphase and lithium dendrite growth during lithium plating/stripping induce poor safety, low Coulombic efficiency, and short span life of lithium metal batteries. Lately, varies of micro/nanostructured lithium metal anodes are proposed to address these issues in lithium metal batteries. With the unique surface, pore, and connecting structures of different nanomaterials, lithium plating/stripping processes have been regulated. Thus the electrochemical properties and lithium morphologies have been significantly improved. These micro/nanostructured lithium metal anodes shed new light on the future applications for lithium metal batteries.

## 1 Introduction

Battery energy storage system has achieved great success in the portable electronics and electronic vehicles.^1^ Among various battery electrode materials, lithium metal is regarded as the “Holy Grail” electrode due to the extra‐high capacity (3860 mA h g^−1^) and the lowest negative electrochemical potential (–3.040 V *vs*. the standard hydrogen electrode).^2^, ^3^ In fact, lithium metal primary batteries have been widely employed in cardiac pacemaker, space exploration, and petroleum prospecting in 1970s.^4^ However, the unstable solid electrolyte interphase (SEI) and uncontrolled dendrite growth during lithium plating/stripping in the rechargeable lithium metal based batteries have prevented their practical applications over the past 40 years. Inhibiting lithium dendrite growth is the rate‐determining step to achieve a safe and efficient operation of lithium metal anodes.^2^ This decides the fate of several promising candidates of energy storage systems for next‐generation energy storage systems, including rechargeable Li‐air batteries,^5^, ^6^ Li‐sulfur batteries,^7^, ^8^, ^9^, ^10^, ^11^ and future Li‐ion batteries.^12^, ^13^, ^14^, ^15^

Compared with the insertion‐host electrode materials (carbon,^16^ oxide,^16^ and silicon^16^, ^17^ etc.), there is no host for lithium metal anode during lithium ion plating, rendering the uncontrollable growth of dendrites. Lithium dendrites are referred as the rod‐like, tree‐like, and moss‐like lithium deposits. The dendrite growth results in two critical problems: (1) Dendrites may impale the membrane to the cathode side, resulting in cell short circuit, thermal runaway, fire, and even explosion. (2) Dendrites are always with a large surface area and can easily react with the electrolyte, which consumes the active materials irreversibly and decreases the efficiency of cells. The “dead Li” resulting from the uneven dissolution of lithium dendrites will further reduce the battery life.^18^

During the past half century, a number of strategies have been proposed to suppress dendrite growth: (1) Stable SEI layer built by electrolyte additives. The SEI layer is in situ formed by the spontaneous reactions between lithium metal and organic electrolyte and can protect lithium metal form the further corrosion.^19^, ^20^, ^21^ The method significantly contributes to the vast applications of Li‐ion batteries. (2) Ex situ (artificial) SEI layer coating. The protective SEI layer can also be built on a lithium metal anode before cell cycling. This ex situ constructing strategy provides a possibility to accurately modify the SEI layer due to researchers' will though the constructing process is a little complicated.^22^, ^23^, ^24^, ^25^ (3) Solid (or polymer) electrolyte. Solid (or polymer) electrolyte avoids the use of the flammable organic electrolyte and improves the cell safety.^26^, ^27^, ^28^ In addition, solid (or polymer) electrolyte is with large modulus that can inhibit dendrite growth in the electrolyte. However, the low room temperature conductivity stops its practical applications in high‐power lithium metal batteries.^29^, ^30^, ^31^

Though many strategies have been proposed to inhibit dendrite growth, lithium metal batteries are still in the infant period. The commercial applications of safe graphite anode in 1990s has sidelined lithium metal anode. However, recently, the high energy density lithium metal batteries have been regarded as among the most promising next‐generation batteries. Compared with the researches in 20^th^ century, the booming development of nanoscience, nanomaterials, and nanoscale characterization renders emerging chance for electrode materials with high efficiency and long cycle life.^25^, ^32^, ^33^, ^34^, ^35^, ^36^ Lithium ion diffusion/deposition heavily depends on the size of electrode materials.^37^ Micro/nanostructured lithium metal anode has become a new powerful strategy to control lithium plating/stripping process and inhibit lithium dendrite growth in lithium metal batteries.^18^, ^38^, ^39^, ^40^, ^41^

Due to the well‐designed structures of all sorts of nanomaterials, micro/nanostructured lithium metal anodes have a lot of properties which are superior to that of the ordinary lithium metal anodes: (1) Tunable surface structures. Nanomaterials always possess a high specific surface area, with which the active surface area in anodes can be increased by several orders of magnitude. One of the most critical issues in lithium metal anode is the interface problem. In the lithium plating process on lithium metal anode, lithium ions should transport from liquid electrolyte side to the solid lithium metal side, in which the transportation, distribution, nucleation, plating reaction are all impacted heavily by the interface properties. The interface also plays the same critical role in the lithium stripping process. By modifying electronic/ionic conductivity, interfacial energy, and other properties of the active surface in micro/nanostructured lithium metal anode, the electrochemical properties such as current density, lithium nucleation overpotential, interface impedance can be fine regulated. Consequently, the lithium plating/stripping process at the interface can be effectively controlled. (2) Pore structures. Micro/nanostructures always possess random 3D pore structures. Hierarchical pore structures which could be precise controlled are also common in well‐designed structures. The clear differences between random/hierarchical pore structure and Li dendrite suppression is not known. The 3D pore structure provides the space for lithium plating and stripping. On one hand, a large pore volume can inhibit large volume change of lithium metal anode during charging/discharging process, because the lithium can plate into and strip out from the micro/nanostructures without the structure change of framework. The stable cycle capacity of lithium plating can be also significantly increased. On the other hand, a high pore volume can offer enough space for electrolyte to permeate. The thickness of the concentration boundary layer can be even thinner, and the polarization voltage and space charge will be decreased. (3) Interconnecting structures. The connecting structure affords the continuous channels for ions to transfer into and out from the micro/nanostructured lithium metal anode. They can control the electronic or ionic transportation and distribution properties in the lithium metal side or in the electrolyte side. During the lithium plating/stripping processes, the lithium ions can easily transfer into or out from the micro/nanostructured anode, and so can the electrons in metal phase. Then the impedance will be decreased and the charge/discharge rate can be remarkably increased. (4) Other electrochemical or physical properties, such as flexibility, shear module, elastic strength, ionic adsorption selectivity, ionic permeation selectivity, etc. With these fine designed micro/nanostructured lithium metal anodes, the reaction rate, active sites, overpotential and other factors of the lithium plating/stripping processes can be investigated and controlled to a great extent. As a result, a dendrite‐free lithium metal anode with high Coulombic efficiency, high cycle stability, as well as low electronic and ionic impedance are highly expected.

**Figure 1:**
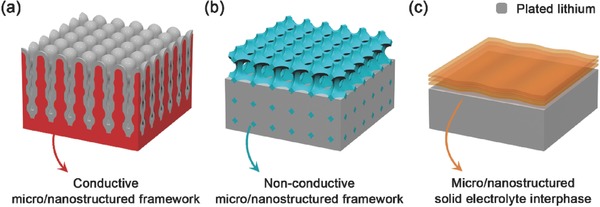
Schematic diagrams of different types of micro/nanostructures. a) Conductive micro/nanostructured framework; b) Non‐conductive micro/nanostructured framework; c) Micro/nanostructured solid electrolyte interphase.

A family of micro/nanostructured lithium metal anode with different properties has been proposed to investigate and regulate the lithium plating/stripping in working rechargeable lithium metal‐based batteries. Here, some typical micro/nanostructured lithium metal anodes are reviewed based on different types of micro/nanostructures: (1) conductive micro/nanostructured framework, (2) non‐conductive micro/nanostructured framework, and (3) micro/nanostructured solid electrolyte interphase (**Figure** **1**). These micro/nanostructures all have different and unique spatial structures including surface, pore, and connecting structures with a variety of physical and chemical properties. The suppression of lithium dendrite growth and improvement of electrochemical performance will be presented and summarized to illustrate the principles and current situations of micro/nanostructured lithium metal anodes. In addition, the issues, challenges, and promising solutions of micro/nanostructured lithium metal anodes will be discussed in detail at the end of this review.

## 2 Conductive Micro/Nanostructured Framework

Lithium ions get electrons at the active surface of the current collector and plate onto it during the lithium plating process, while in the lithium stripping process, lithium atoms loss electrons in turn and are dissolved into the electrolyte. It is obvious that a high electronic conductivity is highly required for the current collector. Therefore, among the various nanomaterials, the conductive nanomaterial is the first one to be considered and widely used as a current collector framework in lithium metal anode. The schematic diagram of conductive micro/nanostructured framework is showed in Figure 1a.

Sand's time model has been proposed to describe the initial nucleation process for lithium dendrite growth as follows:

(1) $$\tau =\pi D{\left(\frac{eC_{0}}{2Jt_{a}}\right)}^{\;2}$$

where the Sand's time τ is the time for ionic concentration at the anode surface dropping to zero and the dendritic lithium starting to grow, *D* is the ambipolar diffusion coefficient, *e* is the electronic charge, *J* is the effective electrode current density, *t*_a_ is the anionic transference number, *C*_0_ is the initial lithium ionic concentration.^42^, ^43^, ^44^, ^45^ As indicated by the Sand's time model, smaller current density *J* leads to the larger Sand's time *τ*. Therefore, the formation of lithium dendrite will be delayed and even inhibited with low effective current density.

The surface structure of the conductive micro/nanostructured framework is used to accommodate the plating of lithium metal due to its high electrical conductivity. With the high specific surface area of conductive nanomaterials, the active surface area for lithium plating is several orders of magnitude higher than ordinary lithium plate anode. As a result, when conducted at the same electrode current density, the local current density and local deposition rate of lithium atoms on the actually effective surface are significantly decreased.^46^ As the Sand's time model discussed above, the Sand's time will be greatly extended, and the lithium dendrite growth is therefore inhibited. The pore structure of the conductive micro/nanostructured framework offers the space for lithium metal plating and the electrolyte permeating as previously mentioned. Besides, some space limited pore structure like layered and stacked structure can physically block the lithium dendrite growth with its high shear module and elastic strength. The connection structure of the conductive micro/nanostructured framework should be strictly interconnected with high electronic conductivity, which is much more critical than other micro/nanostructured framework. The ideal conductive micro/nanostructure should be equipotential. Then lithium ions are therefore uniformly deposited on the framework at the same rate in order to avoid local excess deposition to form lithium dendrites.

Some typical conductive nanomaterials including carbon‐based nanomaterials, metal‐based nanomaterials and porous lithium framework are introduced below.

### 2.1 Carbon‐Based Framework

**Figure 2:**
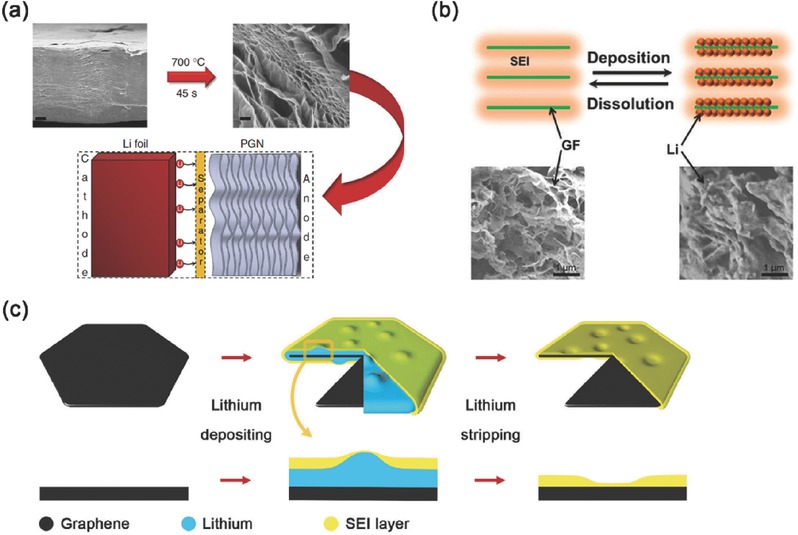
Carbon‐based conductive nanostructured framework with electroplating of lithium. a) Preparation of porous graphene networks and the tests as lithium metal anode in lithium batteries. Reproduced with permission.^47^ Copyright 2014, Nature Publishing Group. b) The lithium plating and stripping on nanostructured graphene framework. Reproduced with permission.^48^ Copyright 2015, American Chemical Society. c) Lithium plating/stripping process on unstacked graphene flake with protective covering SEI layer. Reproduced with permission.^49^

Koratkar and co‐workers described a thermally reduced, free‐standing porous graphene networks as high‐capacity nanostructured lithium metal anode (**Figure** **2**a). They demonstrated that the defects in the graphene lattice acted as seed points that initiate plating of lithium metal within the interior of the porous graphene structure. Such porous graphene structure showed a capacity of 900 mA h g^−1^ (almost threefold higher than conventional graphitic anodes, 372 mA h g^−1^) and an excellent reversibility for over 1000 charge/discharge cycles with the Coulombic efficiencies above 99%.^47^

A distinctive graphene framework structure coated by an in situ formed SEI layer with lithium metal plating in the pores as the nanostructured lithium metal anode is recently proposed by Zhang and co‐workers (Figure 2b). This graphene‐based anode demonstrated a superior dendrite‐inhibition behavior in 70 h of lithiation at 0.5 mA cm^−2^. They proposed that the unblocked ionic pathways and high electronic conductivities of graphene framework in the nanostructured anode led to the rapid transfer of lithium ions through the SEI layer and endowed the graphene‐based anode with an ionic conductivity of 7.81 × 10^−2^ mS cm^−1^.^48^ To further investigate the improvement of employing graphene in lithium metal anode, Zhang's group has constructed a nanostructured lithium metal anode by the unstacked graphene, which exhibited a very high specific surface area, high pore volume and high electrical conductivity (Figure 2c). The local current density of lithium plating sites on graphene‐based anodes was only 4.0 × 10^−5^ mA cm^−2^, ten thousandth of that on routine Cu foil‐based anode at the same electrode current density (0.5 mA cm^−2^). Therefore, the dendrite‐free morphology after lithium plating was received. It was confirmed by controlled experiments and SEM images that the dendrite‐free morphology was induced by the ultralow local current density. Besides, the high pore volume has also endowed such nanostructured lithium metal anode with a high stable cycle capacity of 5 mA h cm^−2^.^49^

Other conductive 3D carbon (e.g. a 3D graphene@Ni scaffold^50^ and carbon nanofiber networks^51^) have also applied to accomplish dendrite‐free Li plating without dendrite formation.

Except nanostructured graphene assemblies, conductive porous carbon film was also proposed to serve as framework for Li plating. Kim's groups described a porous carbon that consisted of amorphous carbon particles as the nanostructured lithium metal anode. This porous carbon film has a high surface area and plenty of inner empty space. The high surface area reduces the effective current density for lithium dissolution and deposition. The empty inner space enables the accommodation of the large volume of dendritic lithium deposits.^52^

**Figure 3:**
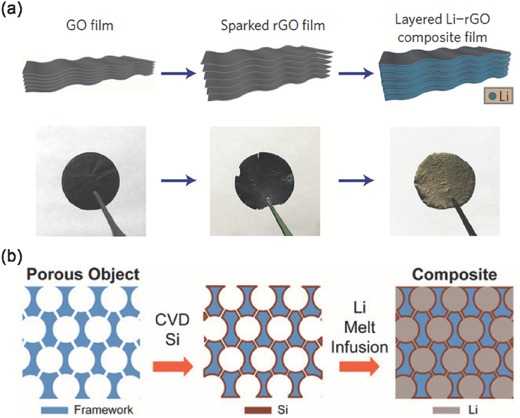
Carbon‐based conductive nanostructured framework with infusion of melted lithium. a) Fabrication of the layered Li‐rGO composite film. Reproduced with permission.^53^ Copyright 2016, Nature Publishing Group. b) Schematic illustration of the design of a Li‐scaffold composite. Reproduced with permission.^54^ Copyright 2016, National Academy of Sciences.

If the surface of conductive framework became lithiophilic due to some surface chemistry modifications, Li ions prefer to uniformly deposit on the conductive surface rather than dendrite growth. This concept was clearly verified by Cui and co‐workers through a uniform layered nanostructured Li‐rGO composite by spark reaction of GO in contact with molten lithium for use as a nanostructured lithium metal anode (**Figure** **3**a). They found that rGO surface functional groups such as carbonyl and alkoxy groups exhibit much stronger binding energy to lithium than the bare graphene, which increased the surface lithiophilicity for lithium intake. Molten lithium infusion was developed for pre‐storing lithium into the lithiophilic interlayer spacing of the sparked rGO. Such anode exhibited low dimension variation (≈20%) during cycling and good mechanical flexibility.^53^ To create a lithium composite nanostructured lithium metal anode, Cui's group has introduced a facile melt‐infusion approach to effectively encapsulate lithium inside a porous carbon scaffold with Si coated lithiophilic layer (Figure 3b). It could deliver a high capacity of 2000 mA h g^−1^ as stable anodes for lithium metal batteries.^54^ They have also reported a graphite‐encapsulated lithium‐metal hybrid anodes with a controlled specific capacity of 744 mA h g^−1^.^55^

Besides, the surface of the nanostructured lithium metal anode can be further modified with artificial nucleation sites. Cui and co‐workers achieved selective deposition and stable encapsulation of lithium metal into hollow carbon nanocapsules with Au nanoparticle seeds inside by heterogeneous seed growth. Such selective deposition and stable encapsulation of lithium metal inhibited lithium dendrite growth and improved cycling with 98% Coulombic efficiency for more than 300 cycles. They explored the nucleation pattern of lithium on various metal substrates and proposed that there was no overpotential to nucleate lithium metal on Au, Ag, Zn, or Mg, with which the plating of lithium could be regulated.^56^

### 2.2 Metal‐Based Framework

**Figure 4:**
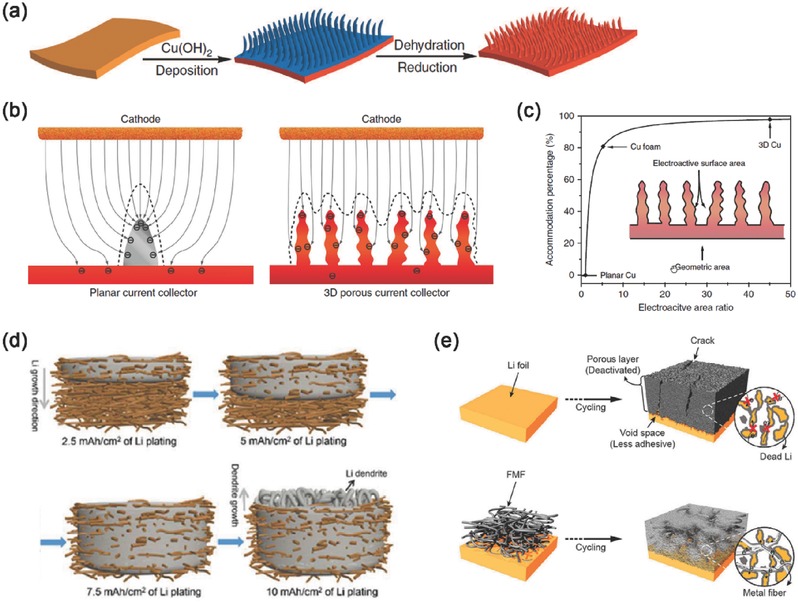
Metal‐based conductive micro/nanostructured framework with simple metal structures. a) Schematic presentation of the procedures to prepare a 3D porous Cu foil from a planar Cu foil. b) Different electrochemical deposition behaviors of lithium metal on planar current collector and 3D current collector. c) Lithium accommodation percentage with electroactive area ratio. a–c) Reproduced with permission.^32^ Copyright 2015, Nature Publishing Group. d) Lithium plating in porous Cu nanowire network. Reproduced with permission.^57^ Copyright 2016, American Chemical Society. e) Schematic diagrams of the evolution of the porous lithium layer for bare lithium and fibrous metal felt/lithium electrode. Reproduced with permission.^58^ Copyright 2016, Nature Publishing Group.

A 3D Cu foil current collector with a submicron skeleton and high electroactive surface area was firstly reported as the metal‐based conductive nanostructured lithium metal anode by Guo and co‐workers (**Figure** **4**a). This lithium metal anode could run for 600 h without short circuit, suppressing the lithium dendrite growth. Because of the submicron structure of the 3D current collector, this lithium anode held a high areal capacity and maintained a high plating/stripping efficiency of 98.5%. They proposed that the percentage of lithium metal deposited inside the 3D structure (η) is generally determined by the electroactive area ratio (*r*, the ratio of electroactive surface area to geometric area of electrode) according to the following formula: (Figure 4b,c)^32^

(2) $$\eta =\frac{r-1}{r}.$$

Recently, Yao, Yu, and co‐workers demonstrated a free‐standing Cu nanowire network current collector which could accommodate the lithium deposition inside the porous nanostructure and suppress the growth of Li dendrites (Figure 4d). 7.5 mA h cm^−2^ of lithium could be plated into the random pore structures of the free‐standing Cu nanowire current collector without lithium dendrite growth. The lithium metal anode could run for 200 cycles with a low and stable voltage hysteresis of 40 mV, and the average Coulombic efficiency of lithium plating/stripping could reach 98.6%.^57^

A fibrous metal felt built by stainless‐steel fibers was introduced as a 3D conductive interlayer between the separator and the lithium metal anode by Kim and co‐workers^58^ to improve the reversibility of the lithium metal anode (Figure 4e). Such conductive microstructured lithium metal anode with random pore structures can be operate at a high current density of 10 mA cm^−2^ with a small polarization of only 30 mV.^58^

**Figure 5:**
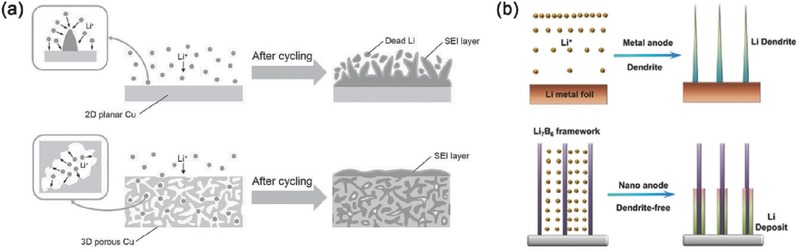
Metal‐based conductive nanostructured framework with lithium alloy or dealloyed metals. a) The structural changes in a conventional planar current collector and in 3D porous current collector built by dealloying. Reproduced with permission.^59^ b) The illustration of routine lithium dendrite formation on the plate lithium metal and lithium deposits on nanostructured anode with metallic lithium contained in fibrous Li_7_B_6_ matrix. Reproduced with permission.^60^

Another 3D porous current collector for lithium metal anodes using chemical dealloying approach was proposed by He, Yang and co‐workers (**Figure** **5**a). The interconnected 3D Cu framework was built by the dissolution of Zn from the Cu‐Zn alloy tape. This 3D current collector with excellent electrical conductivity exhibited a high Coulombic efficiency of 97% for 250 cycles at 0.5 mA cm^−2^, and a long lifespan of 1000 h was achieved.^59^

Zhang and co‐workers^60^ fabricated a conductive 3D nanostructured electrode with metallic Li contained in fibrous Li_7_B_6_ matrix as the lithium metal anodes (Figure 5b). Such lithium metal anode demonstrated stable long cycling performance, high Coulombic efficiency, and an unprecedented cycling life to 2000 cycles beyond plate lithium metal in high‐energy‐density metallic Li‐S batteries.^60^, ^61^

### 2.3 Lithium‐Based Framework

Lithium metal itself can also be designed as conductive micro/nanostructured frameworks in the absence of other components like carbon‐based or metal‐based frameworks. These lithium‐based frameworks are mainly built by porous lithium materials like lithium powder, or surface physical modifications on lithium electrode.

A porous lithium electrode with 25% lithium loading fabricated by lithium powder was proposed by Kong and co‐workers.^62^ It could be cycled over 250 cycles at 5.0 mA cm^−2^ with no lithium dendrite growth.^62^ The electrodes based on coated lithium powder on copper current collector foil exhibited a significantly improved performance and safety compared to lithium foil electrode. The coated lithium powder was in electronic contact with the current collector, and at the same time it could reversibly provide lithium ions and the protective coating was not blocking the lithium ion transfer. During electrode preparation, the coating of the lithium particles was not destroyed. Besides, due to the higher surface area of lithium powder than lithium foil, the coated lithium powder electrodes delivered better Coulombic efficiencies and lower over potentials during cycling.^63^

Bieker's group reported a micro‐needle pre‐treatment technique for lithium metal anode.^64^ Using the micro‐needle roller technique, a large numbers of inverse micro‐needle structure were duplicated onto lithium metal surface, thereby increasing the active surface area. Thus, the charge transfer resistance of the micro‐needle treated lithium metal was reduced, and the cycling performances of cells using the micro‐needle treated lithium metal were improved. A micro‐patterned lithium metal anode has been also proposed by Ryou and Lee's group.^65^ Combined with finite‐element method simulation and experiments, they proved that the micro‐patterned lithium could suppress dendrite growth and improve long‐term cycling stability of lithium metal anodes.

The use of conductive scaffolds provides low local current density in a 3D composite electrode. These porous scaffolds can be well preserve when lithium ions are stripping from the composites. Therefore, the energy chemistry of lithium ion plating/stripping on the porous conductive chemistry is the basis for controllable and safe use of composite lithium anode. It should be noted that highly conductive carbon or metal are non‐polar substrate, while lithium ions are preferred to nuclear on lithiophilic substrates. Therefore, the surface chemistry as well as the dynamic of lithium plating/stripping on a conductive substrate is strongly requested and more quantitative descriptions of current density/electric field/lithium ion diffusions should be considered.

## 3 Non‐Conductive Micro/Nanostructured Framework

The growth of lithium dendrite is impacted heavily by not only the transportation of electrons in solid metal phase, but also the transportation of ions in the liquid electrolyte. It is reported that lithium dendrites will be easily formed at the sites where the surface concentration of lithium ions decreased to near zero, in which means that the migration rate of lithium ions in electrolyte is slower than the plating reaction rate at the surface of lithium metal anode.^44^, ^46^, ^66^, ^67^, ^68^ The migration rate of lithium ions in electrolyte always plays the rate‐determining step during lithium plating/stripping process. Such migration of lithium ions is simultaneously driven by the concentration gradient of the lithium ions and the electric field in the electrolyte phase, and is controlled by the transference number, viscosity, ionic conductivity, and other factors of electrolyte.^42^, ^67^, ^69^ The conductive micro/nanostructured framework mentioned above can adjust the electric field distribution in the electrolyte through its conductive surface structure, and adjust the lithium ion concentration gradient in the electrolyte near the anode surface by reducing the local current density, but the main bulk phase of the electrolyte is lack of strong regulation. Hence, non‐conductive micro/nanostructured framework has been proposed to directly control the concentration distribution, migration rate, and other parameters of lithium ions in electrolyte phase. The schematic diagram of non‐conductive micro/nanostructured framework was explained as Figure 1b.

The surface structure of non‐conductive micro/nanostructured framework plays the most critical role in the regulation of lithium ions in the electrolyte. The non‐conductive nanomaterials whose surface is modified with polar functional groups are widely employed. According to the theoretical calculation and experimental results, the surface polar functional groups are able to adsorb lithium ions in electrolyte and bring about a relatively high local lithium ion concentration. When during lithium plating process, the adsorbed lithium ions near the non‐conductive micro/nanostructured framework can desorb into the bulk phase of electrolyte. Thus the formation of concentration boundary layer can retard. With the sufficient migration rate and supplement of lithium ions, the lithium can then plate on the current collector with a uniform and dendrite‐free morphology. Besides, some polar functional groups that densely distributed on the continuous surface of non‐conductive nanomaterials can increase the transport rate of lithium ions at the surface due to the low surface energy, which is also beneficial for supplying the consuming lithium ions near the electrode surface. The pore structure of non‐conductive micro/nanostructured framework provides the space for electrolyte and plated lithium metal. However, unlike the uniform plating at all over the surface of conductive micro/nanostructured framework (Figure 1a), lithium metal can only plate from the bottom of the non‐conductive micro/nanostructured framework to the top with the increasing thickness of the plated metal anode (Figure 1b). In addition, the connecting structure of such non‐conductive micro/nanostructured framework should ensure the complete permeation of the electrolyte through the pores. The interconnection between non‐conductive nanomaterials is generally unnecessary which is significantly different from the conductive ones.

**Figure 6:**
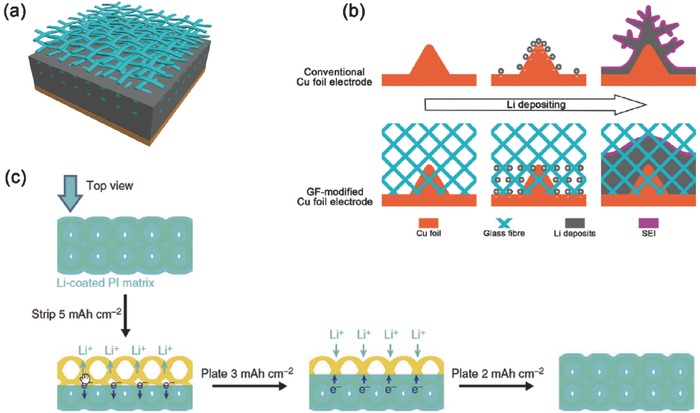
Non‐conductive micro/nanostructured framework. a,b) The schematic diagrams of lithium deposition on 2D Cu foil without and with glass fiber cloth. Reproduced with permission.^69^ Copyright 2003, Electrochemical Society. c) Well‐confined stripping/plating behavior of the lithium‐coated PI matrix. Reproduced with permission.^75^ Copyright 2015, American Chemical Society.

Typical non‐conductive micro/nanostructured lithium metal anodes have been proposed by Zhang's group. Glass fibers with plenty of polar functional groups (Si–O, O–H, O–B) were employed as the interlayer of lithium metal anode and separator (**Figure** **6**a,b). They indicated that the polar functional groups could adsorb considerable lithium ions to compensate the electrostatic interactions and concentration diffusion between lithium ions and protuberances of conventional Cu foil anode. The dendrite‐free deposits were achieved at a high rate of 10.0 mA cm^−2^ and a high lithiation capacity of 2.0 mA h cm^−2^. Besides, stable cycling performance of 500 cycles (170 h) was maintained. These performance improvements were mainly attributed to the uniformly distributed lithium ions near the electrode surface induced by such non‐conductive micro/nanostructure with polar functional groups.^70^ The use of micro/nanostructured electrolyte in which anions are well fixed by SiO_2_/Al_2_O_3_ nanoparticles while Li ion can be uneven diffuse and deposit on the Li metal anode,^71^, ^72^, ^73^, ^74^ which is another effective route to retard the formation of Li dendrites.

Another typical stable cycling of lithium metal anodes by using a chemically inert and electronically insulating oxidized polyacrylonitrile (PAN) nanofiber network as a scaffold has been demonstrated by Cui and co‐workers to guide and confine the deposition of lithium. Homogeneous lithium deposition was embedded inside the nanostructure via attraction forces between the polar functional groups and lithium ions. The Coulombic efficiency could reach 97.4% at a current density of 3.0 mA cm^−2^ and an areal capacity of 1.0 mA h cm^−2^ for more than 120 cycles.^75^ Besides, Cui's group^76^ reported a free‐standing and porous metallic lithium anode by infusing molten lithium into a core‐shell PI(polyimide)‐ZnO matrix (Figure 6c). The heat resistance and chemical stability of the PI fibers guaranteed the structural integrity during lithium coating and battery cycling. Dendrite‐free and minimum‐volume‐change lithium plating/stripping was successfully achieved. Flat voltage profiles and long‐term cycling stability was realized even at a high current density of 5.0 mA cm^−2^. Such non‐conductive polymeric matrix with molten lithium infused was a new strategy for non‐conductive micro/nanostructured lithium metal anodes.

The separator attached with Li metal anode have outstanding role to regulate the stripping/plating of Li metal in a working battery.^77^, ^78^ Nanoprous PI,^79^ polyoxyzole nanofiber membranes,^80^ Janus separator with mesoporous graphene,^81^ graphene/Nafion/PP,^82^ mussel‐inspired polydopamine‐coated separators,^83^ boron‐nitride coated separators,^84^ graphene/PP/Al_2_O_3_ composite separators,^85^ and garnet nanoparticles/polyethylene oxide membrane electrolytes^86^ are also effective to retard the formation of Li dendrites.

The insulating phase modulates the spatial charge distribution and guides the stripping/plating Li ions onto Li metal anode, which is an effective route to regulate the safe use of Li metal. However, the insulating components do not directly participate in the electrochemical reactions. In order to maintain a high‐energy‐density lithium metal battery, the amounts of insulating components in a cell should be minimized. More importantly, the surface chemistry of the insulating and lithiophilic framework is very complicated, which may result in some adverse interactions between them and the organic electrolyte. These interactions may affect the ionic concentration distributions and finally determine the transfer behavior of ions in electrolyte and the lithium plating/stripping process at the electrode surface. Therefore, we call for more attentions on the interfaces between the porous substrate and electrolyte to gain more intrinsic chemistry and physics and therefore to rational design of insulating substrate for safe battery with high energy density.

## 4 Micro/Nanostructured Solid Electrolyte Interphase

It is well known that SEI layer will be formed immediately when highly active lithium metal contacts with liquid electrolytes during charging/discharging processes. However, the in situ formed SEI layer is always unstable in both chemical components and physical structures. Thus, it is easily broken and reformed repeatedly during battery cycles, inducing continuous irreversible consumption of lithium metal and electrolytes, which brings low Coulombic efficiency and shorted battery life. In order to improve the protective nature of SEI layer, nanomaterials are employed as the micro/nanostructured solid electrolyte interphase to inhibit lithium dendrite growth and improve the electrochemical performance of lithium metal anode. The micro/nanostructured solid electrolyte interphases with high shear modulus and high elastic strength are always employed as the physical barrier to block the lithium dendrite growth and make lithium deposits smooth and compact.^87^, ^88^, ^89^ Besides, the micro/nanostructured solid electrolyte interphases with protective chemical components and flexible structures can also act as the “artificial” SEI layer to protect the lithium from side electrochemical reactions with electrolyte. These micro/nanostructures required high ionic conductivity and sufficiently low electronic conductivity, in which way can lithium metal plate underneath the micro/nanostructured SEI layer easily and uniformly with low impedance.^18^, ^89^, ^90^, ^91^ Figure 1c exhibited the schematic diagram of micro/nanostructured solid electrolyte interphases.

In the micro/nanostructured SEI layer that acts mainly as the physical barrier, the connecting structure built by the interconnected pores provides the channels for lithium ions to transport into and out from the current collector. The lithium dendrites cannot grow through these channels and will be blocked by the space limitations. While in the micro/nanostructured SEI layer that aims at improving lithium ion migration and plating performance, the surface structure with lithium ion selective permeability guarantees the high ionic conductivity and protects the lithium metal plated. This kind of micro/nanostructured layer is always with a thickness of nanoscale and a highly flexibility. So that the structure of artificial SEI layer can be reserved well during lithium plating/stripping cycles and play an efficient protection of lithium metal by shielding the electrolyte.

**Figure 7:**
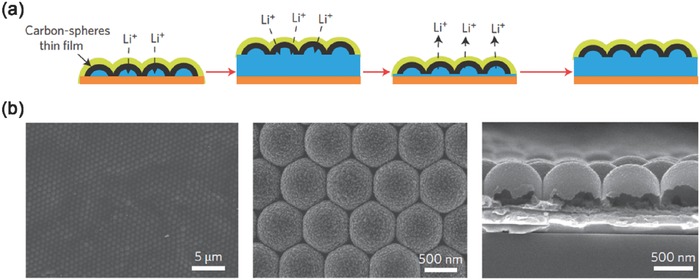
Nanostructured SEI layer. a) Modifying the Cu substrate with a hollow carbon nanospheres layer, with which the volumetric change of the lithium plating process was accommodated. b) SEM images of the carbon‐coated polystyrene nanoparticle array at low/high magnifications, and the cross‐sectional SEM image of the hollow carbon nanospheres. Reproduced with permission.^25^ Copyright 2014, Nature Publishing Group.

A typical nanostructured solid electrolyte interphase layer was proposed by Cui and co‐workers. The artificial SEI was built by a monolayer of interconnected amorphous hollow carbon nanospheres (**Figure** **7**a,b). Such amorphous hollow carbon nanospheres was chemically stable in contact with lithium metal, and has a Young's modulus of 200 GPa, high enough to suppress lithium dendrite growth. The direct measurement on local rate of lithium deposition can identify the role of local mechanical stresses on the deposition of electrolyte at lithium metal anode.^92^ The hollow carbon nanospheres was electrically insulating and ionically conductive. With such interconnected hollow carbon nanospheres, a stable lithium metal anode cycling with uniform dendrite‐free morphology was achieved with the cycling Coulombic efficiency of 99% for more than 150 cycles at 1 mA cm^−2^/1 mA h cm^−2^.^25^

**Figure 8:**
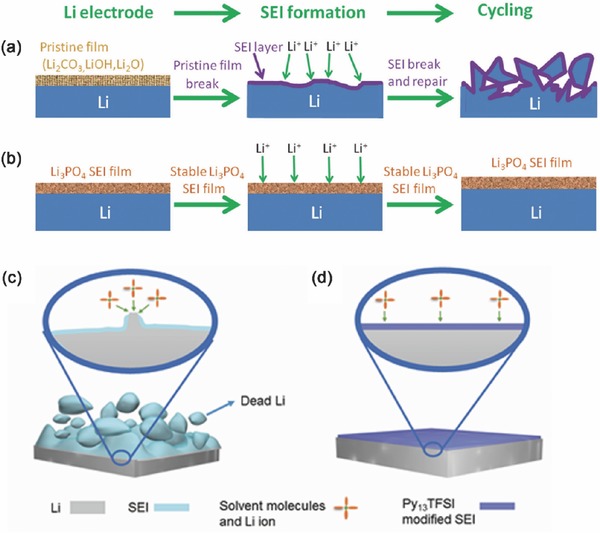
Schematics of the Li anode with artificial SEI. a) Routine Li metal with unstable SEI in organic electrolyte. Reproduced with permission.^97^ Copyright 2016, American Chemical Society. b) Li_3_PO_4_‐modified Li metal anode, which rendering a stable cycling in a working battery.^98^ Schematic diagrams of Li metal structures in Li metal batteries in different electrolytes: (c) ether based electrolyte and (d) optimized hybrid electrolyte. Reproduced with permission.^99^

The replacement of native SEI film of Li metal into artificial SEI layer with high shear modulus is another effective route to protect Li metal anode. In an ether electrolyte, Li_2_CO_3_, LiOH, and Li_2_O can be found in the SEI layer with a porous structure. The composition of pristine SEI on the Li metal is highly depended on the electrolyte (including solvent, salt, and organic electrolyte), operating voltage, and even the period of Li metal interacting with organic electrolyte.^93^, ^94^ The SEI on the Li metal anode becomes unstable when the areal loading of sulfur cathode is high in a Li‐S battery (e.g. more than 3.0 mg cm^−2^).^95^, ^96^, ^97^ When the Li ionic flux is locally enhanced, Li dendrite formation is promoted. The replacement of native SEI into a robust SEI is strongly considered. A recent breakthrough achieved by Guo and co‐workers^98^ demonstrated that an artificial Li_3_PO_4_ SEI layer can retard the formation of Li dendrites and reduce the side reaction between Li metal and the organic electrolyte (**Figure** **8**a,b). The artificial Li_3_PO_4_ SEI is achieved by in situ reaction of polyphosphoric acid and a small amount of moisture in the dimethylsulfoxide solution with Li metal can lead to a rough surface. The effective thickness of artificial Li_3_PO_4_ SEI layer is about 50 nm. It exhibits excellent chemical stability during the Li deposition/dissolution process without breakage. The Li|Li symmetric cell without Li_3_PO_4_ SEI layer delivers a large and irreversible voltage drop at 55 h, which is ascribed to cell failure by dendrite‐induced short circuit. In contrast, the Li‐PPA|PPA‐Li symmetric cell is cycled for over 600 h without short circuiting, indicating that the Li dendrite growth has been significantly restrained. The uniform Li_3_PO_4_ SEI layer effectively restrains Li dendrite growth and reduces the corrosion of bulk Li after 200 cycles in a Li|LiFePO_4_ battery.

It is necessary to indicate that although the artificial SEI layers can reduce the Li dendrite growth and restrain the corrosion of bulk Li, the degeneration of artificial SEI layers is irreversible because the artificial SEI layers cannot regenerate in the electrolyte during cycling. Most recently, Guo and co‐workers^99^ demonstrated a promising Li metal passivation strategy by using hybrid *N*‐propyl‐*N*‐methylpyrrolidinium bis(trifluoromethanesulfonyl)amide (Py_13_TFSI) and ethers electrolyte (Figure 8c,d). The hybrid electrolyte can enhance the stability of SEI layer by Py_13_TFSI modifying in the in situ passivation process.^100^ Because of the synergy between Py_13_TFSI ionic liquid and Li salt concentration, the Li|Cu half cells using hybrid electrolyte show high Coulombic efficiency of Li plating/stripping (99.1% after 360 cycles). The Li dendrite growth and corrosion of Li metal have been efficiently restrained after more than 100 cycles in a Li|LiFePO_4_ battery.

Besides, a bifunctional solid polymer electrolyte with an interpenetrating network of poly(ether‐acrylate) (ipn‐PEA) has been proposed by Guo's group.^101^ Such ipn‐PEA electrolyte suppressed dendrite growth and enhanced kinetics for high‐rate operation of the room‐temperature solid lithium metal batteries.

The use of additives (e.g. Trimethylsilyl chloride,^102^ LiI,^103^ lithium bis(oxalato)borate,^104^ LiNO_3_,^105^, ^106^ CsPF_6_,^107^ LiF,^108^ hexamethylditin,^109^ and lanthanum nitrate^110^) are also benefited for robust nanostructured SEI formation in a working cell. Consequently, the additives can reduce the Li dendrite growth in the Li metal batteries, because the presence of additives can modify the Li surface chemistry and thus strongly affect the Coulombic efficiency of Li plating/stripping.

When Li metal exposed in an ether electrolyte for Li‐S batteries, the soluble polysulfides are dissolved into organic electrolyte. This renders the possibility on the facile use of large sulfur particles and the deposition of inorganic lithium sulfides onto the Li metal. However, the etching of Li anode is widely observed in most research. The addition of LiNO_3_ into the ether electrolyte renders a stable LiSO_x_ and LiNO_y_ in the SEI,^105^ while the continuous consumption of LiNO_3_ is widely detected, especially in large capacity pouch cell. Besides, the gas generation on lithium metal anode in Li‐S batteries is also suppressed when LiNO_3_ is used as electrolyte additives.^106^ When the polysulfides were introduced for a ternary salt electrolyte, a two‐layered SEI was observed on the Li metal. The organic layer with a thickness of 20–50 nm originating from the decomposition of ether electrolyte, while an inorganic layer with LiF, Li_2_S, Li_2_CO_3_ rich components are achieved.^20^, ^48^, ^94^, ^111^ When an electrolyte containing 1.0 m lithium bis(trifluoromethanesulfonyl)imide‐5.0 wt% LiNO_3_‐0.020 m Li_2_S_5_ was applied, Li metal anode is well protected by an in situ formed LiF‐Li_2_S_x_ rich SEI layer.^94^ The LiF‐rich SEI rendered a stable Coulombic efficiency of 95% after 233 cycles for Li‐Cu half cells. A dendrite‐free morphology of Li metal anode is observed under the harsh condition.

The SEI is strongly considered by the materials, chemistry, and engineering communities since the last 40 years. The SEI attached onto the Li metal is always at nanoscale with complex compositions and dynamic sizes. With the rapid development of *operando* and high space/time resolution characterization methods under electrochemical working condition, the evolution of SEI in a working cell is expected to be clearly described in the near future. The use of micro/nanostructured SEI with insulating interface, interconnected ion diffusion pathways, and ultra‐stable surface are verified to be effective to regulate the Li ion plating/stripping. It is highly expected to characterize the role of micro/nanostructured SEI layer in a working pouch cell and provide more mechanistic insights into Li metal anode.

## 5 Conclusions and Perspective

The serious safety concern and poor lifespan of lithium metal anode have hindered the commercial applications of next‐generation energy storage systems with high theoretical energy density (Li‐air batteries, Li‐sulfur batteries, future Li‐ion batteries, etc.). Well‐designed micro/nanostructured lithium metal anodes could effectively improve the safety issue from lithium dendrite growth and enhance the battery cycling performance (including practical energy/power density, charge/discharge efficiency, cycle life, etc.) to further meet the requirements of practical applications. The merits induced by the micro/nanostructured lithium metal anodes can be summarized: (1) Structured Li depositing matrix can realize a low and uniform local current density (using matrixes with high surface area and electronic conductivity), uniform Li ion distribution on the anode surface (using lithiophilic matrixes), reduced Li ion concentration gradient from the bulk electrolyte to the anode surface (using matrixes with short ion diffusing routes), thus achieving a dendrite‐free Li deposits and improving the safety performance of Li metal based batteries. (2) During Li plating/stripping, structured lithium anode with an inert matrix can effectively relieve the volume expanding/shrinking even under 100% DOD, while the routine Li metal anode goes through an infinite volume change under 100% DOD. Stability in volume is critically important for a practical battery. (3) With the well‐designed structure of Li metal anode, Li ions and electron can diffuse quickly to achieve high power performance. (4) Structured SEI can maintain high stability during long‐term cycling to protect the corrosion of Li metal from the electrolyte, thus obtaining a high Coulombic efficiency. When conducting the strategies of micro/nanostructured lithium metal anode into a full cell, high and stable cycling capacity, high Coulombic efficiency, and long lifespan are expected to be achieved at high charging/discharging rate.

However, under the current level of nanomaterials and nanotechnologies, micro/nanostructured lithium metal anodes are still not up to the challenges to perfectly solve all the problems in lithium metal anodes. A vast variety of issues of both the original lithium metal anodes and newly introduced nanomaterials are still requested to be well addressed. (1) Low initial cycling efficiency induced by high specific surface area. Although the high specific surface area brings about low local current density, low local deposition rate, and inhibits lithium dendrites, it also significantly increased the contact interface area of the plated lithium metal and electrolyte. Thus a mass of SEI layer will form to cover the high interface area, which results in a considerable consumption of lithium metal and electrolyte in the initial cycles. As a result, the Coulombic efficiency in micro/nanostructured lithium metal anode is usually at a low level for the initial one or few cycles. Such low lithium utilization efficiency is very lethal for micro/nanostructure applications in lithium metal anode. (2) Low energy density of the whole battery. The micro/nanostructured materials are with a low packing density and occupy a large volume of space in batteries. Thus the volumetric energy density cannot be high enough to meet the requirements of commercial applications. (3) Unstable structure during prolonged cycles. The microstructure and electrochemical properties of some fine designed micro/nanostructures will change in the charge‐discharge process. The micro/nanostructures will not perform their original designed functions in regulating the behavior of lithium plating/stripping during long periods of cycling. Much higher stability in structure and electrochemical properties is required.

Some promising strategies to address these challenges of micro/nanostructured lithium metal anode have also been proposed and investigated by the researchers, with more strategies still needed to be inspected. (1) Regulating the SEI layer by modifing the liquid electrolyte. In order to reduce the consumption of lithium metal and electrolyte in the formation of SEI layer on the micro/nanostructures with high specific surface area as much as possible, the SEI layer with high stability, flexibility, and thin thickness is required. By modifying the components of liquid electrolyte, or introducing additives into the electrolyte, the utilization of lithium metal in some micro/nanostructured lithium metal anodes has been successfully improved with high Coulombic efficiencies for hundreds of cycles. (2) Hybrid micro/nanostructure of different nanomaterials. Hybrid micro/nanostructured lithium metal anodes can often possess excellent electrochemical properties and structural properties from a variety of nanomaterials. For instance, the conductive carbon materials can enhance the interconnection of micro/nanostructures with high electric conductivity; the non‐conductive polymer fibers with high electrochemical inertness can increase the structural stability of micro/nanostructures in prolong cycles. In addition to the connection structure, the surface structure can also be modified with composite nanomaterials. The Au nanoparticles for instance were introduced to regulate the nucleation behavior of lithium metal on the surface of carbon nanomaterials. (3) Composite with solid‐state electrolyte. The components and properties of SEI layer formed in liquid electrolyte are difficult to be investigated and regulated due to its complexity and unstable nature. To avoid the problems induced by the liquid electrolyte, the solid‐state electrolyte has been widely employed in lithium ion batteries. Hence, the composite micro/nanostructure of nanomaterials and solid‐state electrolyte is commonly believed to be the promising strategy to address the challenges in lithium metal anode. On one hand, the solid‐state electrolyte can deal with the issue of unstable SEI layer in liquid electrolyte, which can significantly increase the utilization of lithium metal. On the other hand, the nanomaterials can in turn address the surface contact issues and low ionic conductivity faced by the solid‐state electrolyte. Such composite micro/nanostructures lead a new route to overcome the challenges in lithium metal anode.

Finally, micro/nanostructured lithium metal anodes have brought remarkable improvements in both electrochemical properties of lithium metal batteries and the stable lithium plating/stripping process with dendrite‐free morphology. However, these micro/nanostructured electrodes were not mature and perfect enough to address the issues in lithium metal anodes. Future researches of micro/nanostructured lithium metal anode on its electrochemical mechanisms, characterizations, and regulations are still highly required to understand and design better electrodes. New forms of well‐designed micro/nanostructures, especially the composite micro/nanostructures are highly expected for their promising properties in both safety and electrochemistry to face the challenges in high‐energy‐density lithium metal batteries.

## References

[1] M. Armand , J. M. Tarascon , Nature 2008, 451, 652. 18256660 10.1038/451652a

[2] W. Xu , J. L. Wang , F. Ding , X. L. Chen , E. Nasybutin , Y. H. Zhang , J. G. Zhang , Energy Environ. Sci. 2014, 7, 513.

[3] X.‐B. Cheng , Q. Zhang , J. Mater. Chem. A 2015, 3, 7207.

[4] E. C. Evarts , Nature 2015, 526, S93. 26509953 10.1038/526S93a

[5] P. G. Bruce , S. A. Freunberger , L. J. Hardwick , J. M. Tarascon , Nat. Mater. 2012, 11, 19. 10.1038/nmat3191 22169914

[6] Z. L. Wang , D. Xu , J. J. Xu , X. B. Zhang , Chem. Soc. Rev. 2014, 43, 7746. 24056780 10.1039/c3cs60248f

[7] Y. X. Yin , S. Xin , Y. G. Guo , L. J. Wan , Angew. Chem. Int. Ed. 2013, 52, 13186. 10.1002/anie.201304762 24243546

[8] A. Manthiram , S.‐H. Chung , C. Zu , Adv. Mater. 2015, 27, 1980. 25688969 10.1002/adma.201405115

[9] Z. W. Seh , Y. Sun , Q. Zhang , Y. Cui , Chem. Soc. Rev. 2016, 45, 5605. 27460222 10.1039/c5cs00410a

[10] Y. X. Yin , H. R. Yao , Y. G. Guo , Chinese Phys. B 2016, 25, 018801.

[11] J. Liang , Z.‐H. Sun , F. Li , H.‐M. Cheng , Energy Storage Mater. 2016, 2, 76.

[12] R. Chen , R. Luo , Y. Huang , F. Wu , L. Li , Adv. Sci. 2016, 3, 1600051. 10.1002/advs.201600051 PMC5096057 27840796

[13] D. Wang , W. Zhang , W. Zheng , X. Cui , T. Rojo , Q. Zhang , Adv. Sci. 2017, 4, 1600168. 10.1002/advs.201600168 PMC5238744 28105393

[14] L. Yue , J. Ma , J. Zhang , J. Zhao , S. Dong , Z. Liu , G. Cui , L. Chen , Energy Storage Mater. 2016, 5, 139.

[15] H. Li , Z. X. Wang , L. Q. Chen , X. J. Huang , Adv. Mater. 2009, 21, 4593.

[16] J. M. Tarascon , M. Armand , Nature 2001, 414, 359. 11713543 10.1038/35104644

[17] N. Liu , Z. D. Lu , J. Zhao , M. T. McDowell , H. W. Lee , W. T. Zhao , Y. Cui , Nat. Nanotechnol. 2014, 9, 187. 24531496 10.1038/nnano.2014.6

[18] X.‐B. Cheng , R. Zhang , C.‐Z. Zhao , F. Wei , J.‐G. Zhang , Q. Zhang , Adv. Sci. 2016, 3, 1500213. 10.1002/advs.201500213 PMC5063117 27774393

[19] R. R. Miao , J. Yang , X. J. Feng , H. Jia , J. L. Wang , Y. N. Nuli , J. Power Sources 2014, 271, 291.

[20] C.‐Z. Zhao , X.‐B. Cheng , R. Zhang , H.‐J. Peng , J.‐Q. Huang , R. Ran , Z.‐H. Huang , F. Wei , Q. Zhang , Energy Storage Mater. 2016, 3, 77.

[21] C. Zu , A. Dolocan , P. Xiao , S. Stauffer , G. Henkelman , A. Manthiram , Adv. Energy Mater. 2016, 6, 1501933.

[22] G. Ma , Z. Wen , M. Wu , C. Shen , Q. Wang , J. Jin , X. Wu , Chem. Commun. 2014, 50, 14209. 10.1039/c4cc05535g 25285341

[23] D. J. Lee , H. Lee , Y. J. Kim , J. K. Park , H. T. Kim , Adv. Mater. 2016, 28, 857. 26627981 10.1002/adma.201503169

[24] Q.‐C. Liu , J.‐J. Xu , S. Yuan , Z.‐W. Chang , D. Xu , Y.‐B. Yin , L. Li , H.‐X. Zhong , Y.‐S. Jiang , J.‐M. Yan , X.‐B. Zhang , Adv. Mater. 2015, 27, 5241. 26265402 10.1002/adma.201501490

[25] G. Y. Zheng , S. W. Lee , Z. Liang , H. W. Lee , K. Yan , H. B. Yao , H. T. Wang , W. Y. Li , S. Chu , Y. Cui , Nat. Nanotechnol. 2014, 9, 618. 25064396 10.1038/nnano.2014.152

[26] J. Gao , Y. S. Zhao , S. Q. Shi , H. Li , Chinese Phys. B 2016, 25, 018211.

[27] K. Fu , Y. H. Gong , J. Q. Dai , A. Gong , X. G. Han , Y. G. Yao , C. W. Wang , Y. B. Wang , Y. N. Chen , C. Y. Yan , Y. J. Li , E. D. Wachsman , L. B. Hu , Proc. Natl. Acad. Sci. USA 2016, 113, 7094.27307440

[28] W. Luo , Y. Gong , Y. Zhu , K. K. Fu , J. Dai , S. D. Lacey , C. Wang , B. Liu , X. Han , Y. Mo , E. D. Wachsman , L. Hu , J. Am. Chem. Soc. 2016, 138, 12258. 27570205 10.1021/jacs.6b06777

[29] E. Rangasamy , Z. Liu , M. Gobet , K. Pilar , G. Sahu , W. Zhou , H. Wu , S. Greenbaum , C. Liang , J. Am. Chem. Soc. 2015, 137, 1384. 25602621 10.1021/ja508723m

[30] Q. Ma , H. Zhang , C. Zhou , L. Zheng , P. Cheng , J. Nie , W. Feng , Y.‐S. Hu , H. Li , X. Huang , L. Chen , M. Armand , Z. Zhou , Angew. Chem. Int. Ed. 2016, 55, 2521. 10.1002/anie.201509299 26840215

[31] D. Zhou , Y.‐B. He , R. Liu , M. Liu , H. Du , B. Li , Q. Cai , Q.‐H. Yang , F. Kang , Adv. Energy Mater. 2015, 5, 1500353.

[32] C. P. Yang , Y. X. Yin , S. F. Zhang , N. W. Li , Y. G. Guo , Nat. Commun. 2015, 6, 8058. 26299379 10.1038/ncomms9058 PMC4560781

[33] F. Ding , W. Xu , G. L. Graff , J. Zhang , M. L. Sushko , X. L. Chen , Y. Y. Shao , M. H. Engelhard , Z. M. Nie , J. Xiao , X. J. Liu , P. V. Sushko , J. Liu , J. G. Zhang , J. Am. Chem. Soc. 2013, 135, 4450. 23448508 10.1021/ja312241y

[34] Y. G. Guo , J. S. Hu , L. J. Wan , Adv. Mater. 2008, 20, 2878.

[35] Y. Sun , N. Liu , Y. Cui , Nat. Energy 2016, 1, 16071.

[36] N. Mahmood , Y. Hou , Adv. Sci. 2014, 1, 1400012. 10.1002/advs.201400012 PMC5115266 27980896

[37] H. R. Yao , Y. X. Yin , Y. G. Guo , Chinese Phys. B 2016, 25, 018203.

[38] M. D. Tikekar , S. Choudhury , Z. Tu , L. A. Archer , Nat. Energy 2016, 1, 16114.

[39] R. G. Cao , W. Xu , D. P. Lv , J. Xiao , J. G. Zhang , Adv. Energy Mater. 2015, 5, 1402273.

[40] K. Zhang , G.‐H. Lee , M. Park , W. Li , Y.‐M. Kang , Adv. Energy Mater. 2016, 6, 1600811.

[41] Q. Liu , C. Du , B. Shen , P. Zuo , X. Cheng , Y. Ma , G. Yin , Y. Gao , RSC Adv. 2016, 6, 88683.

[42] C. Brissot , M. Rosso , J. N. Chazalviel , P. Baudry , S. Lascaud , Electrochim. Acta 1998, 43, 1569.

[43] C. Brissot , M. Rosso , J. N. Chazalviel , S. Lascaud , J. Power Sources 2001, 94, 212.

[44] M. Rosso , T. Gobron , C. Brissot , J. N. Chazalviel , S. Lascaud , J. Power Sources 2001, 97–98, 804.

[45] R. Akolkar , J. Power Sources 2013, 232, 23.

[46] J. N. Chazalviel , Phys. Rev. A 1990, 42, 7355. 9904050 10.1103/physreva.42.7355

[47] R. Mukherjee , A. V. Thomas , D. Datta , E. Singh , J. Li , O. Eksik , V. B. Shenoy , N. Koratkar , Nat. Commun. 2014, 5, 3710. 24751669 10.1038/ncomms4710

[48] X.‐B. Cheng , H.‐J. Peng , J.‐Q. Huang , R. Zhang , C.‐Z. Zhao , Q. Zhang , ACS Nano 2015, 9, 6373. 26042545 10.1021/acsnano.5b01990

[49] R. Zhang , X.‐B. Cheng , C.‐Z. Zhao , H.‐J. Peng , J.‐L. Shi , J.‐Q. Huang , J. Wang , F. Wei , Q. Zhang , Adv. Mater. 2016, 28, 2155. 26754639 10.1002/adma.201504117

[50] K. Xie , W. Wei , K. Yuan , W. Lu , M. Guo , Z. Li , Q. Song , X. Liu , J.‐G. Wang , C. Shen , ACS Appl. Mater. Interfaces 2016, 8, 26091. 27617633 10.1021/acsami.6b09031

[51] A. Zhang , X. Fang , C. Shen , Y. Liu , C. Zhou , Nano Res. 2016, 9, 3428.

[52] H.‐K. Kang , S.‐G. Woo , J.‐H. Kim , S.‐R. Lee , Y.‐J. Kim , Electrochim. Acta 2015, 176, 172.

[53] D. Lin , Y. Liu , Z. Liang , H.‐W. Lee , J. Sun , H. Wang , K. Yan , J. Xie , Y. Cui , Nat. Nanotechnol. 2016, 11, 626. 26999479 10.1038/nnano.2016.32

[54] Z. Liang , D. Lin , J. Zhao , Z. Lu , Y. Liu , C. Liu , Y. Lu , H. Wang , K. Yan , X. Tao , Y. Cui , Proc. Natl. Acad. Sci. USA 2016, 113, 2862. 26929378 10.1073/pnas.1518188113 PMC4801240

[55] Y. Sun , G. Zheng , Z. W. Seh , N. Liu , S. Wang , J. Sun , H. R. Lee , Y. Cui , Chem 2016, 1, 287.

[56] K. Yan , Z. Lu , H.‐W. Lee , F. Xiong , P.‐C. Hsu , Y. Li , J. Zhao , S. Chu , Y. Cui , Nat. Energy 2016, 1, 16010.

[57] L. L. Lu , J. Ge , J. N. Yang , S. M. Chen , H. B. Yao , F. Zhou , S. H. Yu , Nano Lett. 2016, 16, 4431. 27253417 10.1021/acs.nanolett.6b01581

[58] H. Lee , J. Song , Y.‐J. Kim , J.‐K. Park , H.‐T. Kim , Sci. Rep. 2016, 6, 30830. 27484160 10.1038/srep30830 PMC4971473

[59] Q. Yun , Y. B. He , W. Lv , Y. Zhao , B. Li , F. Kang , Q. H. Yang , Adv. Mater. 2016, 28, 6932. 27219349 10.1002/adma.201601409

[60] X. B. Cheng , H. J. Peng , J. Q. Huang , F. Wei , Q. Zhang , Small 2014, 10, 4257. 25074801 10.1002/smll.201401837

[61] X. Zhang , W. Wang , A. Wang , Y. Huang , K. Yuan , Z. Yu , J. Qiu , Y. Yang , J. Mater. Chem. A 2014, 2, 11660.

[62] S. K. Kong , B. K. Kim , W. Y. Yoon , J. Electrochem. Soc. 2012, 159, A1551.

[63] J. Heine , S. Kruger , C. Hartnig , U. Wietelmann , M. Winter , P. Bieker , Adv. Energy Mater. 2014, 4, 1400406.

[64] M. H. Ryou , Y. M. Lee , Y. J. Lee , M. Winter , P. Bieker , Adv. Funct. Mater. 2015, 25, 834.

[65] J. Park , J. Jeong , Y. Lee , M. Oh , M.‐H. Ryou , Y. M. Lee , Adv. Mater. Interfaces 2016, 3, 1600140.

[66] T. A. Witten , L. M. Sander , Phys. Rev. B 1983, 27, 5686.

[67] K. Nishikawa , T. Mori , T. Nishida , Y. Fukunaka , M. Rosso , J. Electroanal. Chem. 2011, 661, 84.

[68] B. Scharifker , G. Hills , Electrochim. Acta 1983, 28, 879.

[69] C. Monroe , J. Newman , J. Electrochem. Soc. 2003, 150, A1377.

[70] X.‐B. Cheng , T.‐Z. Hou , R. Zhang , H.‐J. Peng , C.‐Z. Zhao , J.‐Q. Huang , Q. Zhang , Adv. Mater. 2016, 28, 2888. 26900679 10.1002/adma.201506124

[71] Y. Y. Lu , M. Tikekar , R. Mohanty , K. Hendrickson , L. Ma , L. A. Archer , Adv. Energy Mater. 2015, 5, 1402073.

[72] S. Choudhury , A. Agrawal , S. Y. Wei , E. Jeng , L. A. Archer , Chem. Mater. 2016, 28, 2147.

[73] M. D. Tikekar , L. A. Archer , D. L. Koch , Sci. Adv. 2016, 2, e1600320. 27453943 10.1126/sciadv.1600320 PMC4956395

[74] S. Choudhury , R. Mangal , A. Agrawal , L. A. Archer , Nat. Commun. 2015, 6, 10101. 26634644 10.1038/ncomms10101 PMC4686773

[75] Z. Liang , G. Zheng , C. Liu , N. Liu , W. Li , K. Yan , H. Yao , P.‐C. Hsu , S. Chu , Y. Cui , Nano Lett. 2015, 15, 2910. 25822282 10.1021/nl5046318

[76] Y. Liu , D. Lin , Z. Liang , J. Zhao , K. Yan , Y. Cui , Nat. Commun. 2016, 7, 10992. 26987481 10.1038/ncomms10992 PMC4802050

[77] Z. Y. Tu , P. Nath , Y. Y. Lu , M. D. Tikekar , L. A. Archer , Accounts. Chem. Res. 2015, 48, 2947. 10.1021/acs.accounts.5b00427 26496667

[78] J.‐Q. Huang , Q. Zhang , F. Wei , Energy Storage Mater. 2015, 1, 127.

[79] D. C. Lin , D. Zhuo , Y. Y. Liu , Y. Cui , J. Am. Chem. Soc. 2016, 138, 11044. 27498838 10.1021/jacs.6b06324

[80] X. M. Hao , J. Zhu , X. Jiang , H. T. Wu , J. S. Qiao , W. Sun , Z. H. Wang , K. N. Sun , Nano Lett. 2016, 16, 2981. 27105287 10.1021/acs.nanolett.5b05133

[81] H. J. Peng , D. W. Wang , J. Q. Huang , X. B. Cheng , Z. Yuan , F. Wei , Q. Zhang , Adv. Sci. 2016, 3, 1500268. 10.1002/advs.201500268 PMC5054863 27774384

[82] T. Z. Zhuang , J. Q. Huang , H. J. Peng , L. Y. He , X. B. Cheng , C. M. Chen , Q. Zhang , Small 2016, 12, 381. 26641415 10.1002/smll.201503133

[83] M. H. Ryou , D. J. Lee , J. N. Lee , Y. M. Lee , J. K. Park , J. W. Choi , Adv. Energy Mater. 2012, 2, 645.

[84] W. Luo , L. H. Zhou , K. Fu , Z. Yang , J. Y. Wan , M. Manno , Y. G. Yao , H. L. Zhu , B. Yang , L. B. Hu , Nano Lett. 2015, 15, 6149. 26237519 10.1021/acs.nanolett.5b02432

[85] R. S. Song , R. P. Fang , L. Wen , Y. Shi , S. G. Wang , F. Li , J. Power Sources 2016, 301, 179.

[86] J. Zhang , N. Zhao , M. Zhang , Y. Li , P. K. Chu , X. Guo , Z. Di , X. Wang , H. Li , Nano Energy 2016, 28, 447.

[87] C. Monroe , J. Newman , J. Electrochem. Soc. 2004, 151, A880.

[88] C. Monroe , J. Newman , J. Electrochem. Soc. 2005, 152, A396.

[89] K. Xu , Chem. Rev. 2014, 114, 11503. 25351820 10.1021/cr500003w

[90] L. Yue , J. Ma , J. Zhang , J. Zhao , S. Dong , Z. Liu , G. Cui , L. Chen , Energy Storage Mater. 2016, 5, 139.

[91] M. Park , X. C. Zhang , M. D. Chung , G. B. Less , A. M. Sastry , J. Power Sources 2010, 195, 7904.

[92] K. J. Harry , K. Higa , V. Srinivasan , N. P. Balsara , J. Electrochem. Soc. 2016, 163, A2216.

[93] S. Z. Xiong , K. Xie , Y. Diao , X. B. Hong , J. Power Sources 2014, 246, 840.

[94] C. Yan , X.‐B. Cheng , C.‐Z. Zhao , J.‐Q. Huang , S.‐T. Yang , Q. Zhang , J. Power Sources 2016, 327, 212.

[95] X.‐B. Cheng , C. Yan , J.‐Q. Huang , P. Li , L. Zhu , L. Zhao , Y. Zhang , W. Zhu , S.‐T. Yang , Q. Zhang , Energy Storage Mater. 2017, 6, 18.

[96] L. Qie , C. X. Zu , A. Manthiram , Adv. Energy Mater. 2016, 6, 1502459.

[97] W. C. Du , Y. X. Yin , X. X. Zeng , J. L. Shi , S. F. Zhang , L. J. Wan , Y. G. Guo , ACS Appl. Mater. Interfaces 2016, 8, 3584. 26378622 10.1021/acsami.5b07468

[98] N. W. Li , Y. X. Yin , C. P. Yang , Y. G. Guo , Adv. Mater. 2016, 28, 1853. 26698171 10.1002/adma.201504526

[99] N. W. Li , Y. X. Yin , J. Y. Li , C. H. Zhang , Y. G. Guo , Adv. Sci. 2017, 4, 1600400. 10.1002/advs.201600400 PMC5323882 28251057

[100] A. Basile , A. I. Bhatt , A. P. O'Mullane , Nat. Commun. 2016, 7, 11794. 10.1038/ncomms11794 PMC4909938 27292652

[101] X.‐X. Zeng , Y.‐X. Yin , N.‐W. Li , W.‐C. Du , Y.‐G. Guo , L.‐J. Wan , J. Am. Chem. Soc. 2016, 138, 15825. 27960330 10.1021/jacs.6b10088

[102] M. F. Wu , Z. Y. Wen , J. Jin , B. V. R. Chowdari , ACS Appl. Mater. Interfaces 2016, 8, 16386. 27269577 10.1021/acsami.6b02612

[103] Y. Y. Lu , Z. Y. Tu , L. A. Archer , Nat. Mater. 2014, 13, 961. 25108613 10.1038/nmat4041

[104] H. Xiang , P. Shi , P. Bhattacharya , X. Chen , D. Mei , M. E. Bowden , J. Zheng , J.‐G. Zhang , W. Xu , J. Power Sources 2016, 318, 170.

[105] D. Aurbach , E. Pollak , R. Elazari , G. Salitra , C. S. Kelley , J. Affinito , J. Electrochem. Soc. 2009, 156, A694.

[106] A. Jozwiuk , B. B. Berkes , T. Weiß , H. Sommer , J. Janek , T. Brezesinski , Energy Environ. Sci. 2016, 9, 2603.

[107] Y. H. Zhang , J. F. Qian , W. Xu , S. M. Russell , X. L. Chen , E. Nasybulin , P. Bhattacharya , M. H. Engelhard , D. H. Mei , R. G. Cao , F. Ding , A. V. Cresce , K. Xu , J. G. Zhang , Nano Lett. 2014, 14, 6889. 25419865 10.1021/nl5039117

[108] S. Choudhury , L. A. Archer , Adv. Electronic Mater. 2016, 2, 1500246.

[109] M. Wu , J. Jin , Z. Wen , RSC Adv. 2016, 6, 40270.

[110] S. Liu , G. R. Li , X. P. Gao , ACS Appl. Mater. Interfaces 2016, 8, 7783. 26981849 10.1021/acsami.5b12231

[111] W. Li , H. Yao , K. Yan , G. Zheng , Z. Liang , Y. M. Chiang , Y. Cui , Nat. Commun. 2015, 6, 7436. 26081242 10.1038/ncomms8436

